# Factors associated with low handgrip strength in older people: data of the Study of Chronic Diseases (Edoc-I)

**DOI:** 10.1186/s12889-020-08504-z

**Published:** 2020-03-26

**Authors:** Cledir de Araújo Amaral, Thatiana Lameira Maciel Amaral, Gina Torres Rego Monteiro, Maurício Teixeira Leite de Vasconcellos, Margareth Crisóstomo Portela

**Affiliations:** 1 Federal Institute of Education, Science and Technology of Acre, Rio Branco, AC Brazil; 2 Center for Health Sciences and Sports, Federal University of Acre, Rio Branco, AC Brazil; 3 Department of Epidemiological and Quantitative Methods in Health, National School of Public Health Sergio Arouca, Oswaldo Cruz Foundation, Rio de Janeiro, RJ Brazil; 4 National School of Statistical Sciences, Brazilian Institute of Geography and Statistics, Rio de Janeiro, RJ Brazil; 5 Department of Health Administration and Planning, National School of Public Health Sergio Arouca, Oswaldo Cruz Foundation, Rio de Janeiro, RJ Brazil

**Keywords:** Hand strength, Insomnia, Diabetes mellitus, Anemia, Dependent elderly, Elderly’s health, Epidemiological survey

## Abstract

**Background:**

Handgrip strength (HGS) is an important health biomarker whose low scores have been shown to be associated with the morbimortality. This study aimed to analyze the factors associated with low HGS in older people in Rio Branco, Acre, Brazil.

**Methods:**

The study was carried out with data from the Study of Chronic Diseases (EDOC-I) – Older People, a cross-sectional household PAPI probability sample survey performed with 1016 people aged over 60 residing in Rio Branco in 2014. The low HGS was defined by the 20th percentile of the maximum HGS by sex and age group. Associations between variables of health status (psychological and physical) and low HGS, by sex, were estimated using logistic regression, expressed by adjusted ORs (aOR).

**Results:**

Older individuals had lower median HGS than younger individuals (− 6.0 kg among men and − 2.6 kg among women). Women aged over 80 had, on average, the lower quintile of HGS compared to women of the previous age groups. Factors independently associated with low HGS in men and women, respectively, were low weigh in body mass index [(aOR = 2.80; 95%CI: 1.19, 6.61) and (aOR = 2.61; 95%CI: 1.46, 4.66)], anemia [(aOR = 4.15; 95%CI: 2.09, 8.21) and (aOR = 1.80; 95%CI: 1.06, 3.06)] and diabetes as a risk factor in men (aOR 1.95; 95%CI: 1.00, 3.81). There was a higher chance of low HGS in men with partners (aOR = 2.44; 95%CI: 1.32, 4.51), smokers or former smokers (aOR = 3.25; 95%CI: 1.25, 8.44), with current self-assessment of health worse than the 12 previous months (aOR = 2.21; 95%CI: 1.14, 4.30) and dependence in activities of daily living (aOR = 2.92; 95%CI: 1.35, 6.30). Only among women, there was an increased chance of low HGS associated with altered waist-to-hip ratio (aOR = 1.79; 95%CI: 1.02, 3.12), insomnia (aOR = 1.83; 95%CI: 1.10, 3.03) and physical activity from displacement/occupation (aOR = 1.75; 95%CI: 1.08, 2.84).

**Conclusion:**

Factors associated with low HGS are not the same between sexes, and the inclusion of HGS as a component of health assessment seems to be a promising strategy for disease prevention and health promotion.

## Background

Population aging is a worldwide phenomenon accompanied by the incidence of physical limitations that result in decreased quality of life and increased health care costs. These limitations pose higher risks of falls, institutionalization, comorbidities, and premature mortality. The loss of muscle mass and strength contributes significantly to physical incapacities in aging [1].

Musculoskeletal functionality plays an important role in health and disease, and it is influenced by age [2]. In the aging process, the capacity of the locomotor system and the secretory function of myosin, which act on the metabolism, is reduced, as well as the function of the muscular tissue and other tissues and organs [3], causing a reduction of muscle strength. The evaluation of such strength gains importance. Therefore, as an indicator of muscular quality and functionality, low muscle strength represents a significant public health problem [4].

Handgrip strength (HGS) is a means of measuring muscle strength that has been used to evaluate important health outcomes in older people [5]. Low HGS is associated with sarcopenia and plays an important role in the definition of the frailty phenotype [6]. It is also associated with falls, reduced functional autonomy, and musculoskeletal complaints [7]. The association between reduced HGS and the presence of depression [8], insomnia, diabetes, hypertension, cardiovascular diseases, multimorbidity [4, 7], and mortality [1] has also been demonstrated.

Further, a relation of HGS with indicators of social inequalities, demographic and behavioral conditions has been reported [4].

HGS as a means of tracking diseases and health problems favors the adoption of disease protection and health promotion actions in order to minimize the impacts of morbidity and mortality of the population. However, more knowledge needs to be consolidated to understand the relationship between HGS and morbidity, considering the possibility of these relationships to vary across different population groups. Only then, its use as a health biomarker can be ratified, and conditions of its applicability can be consistently defined.

In order to contribute to the construction of this knowledge, the objective of this study was to analyze the factors associated with low handgrip strength in older people in the City of Rio Branco, Acre, the northern region of Brazil.

## Methods

### Study setting

This is a secondary analysis of data from the Study of Chronic Diseases in Older People (*Estudo das Doenças Crônicas em Idosos* – EDOC-I), a household survey conducted between April and September 2014, with older people (60 years old or older) living in urban and rural areas of Rio Branco City, Acre State, Brazil. Rio Branco is located in the Western Brazilian Amazon. The Brazilian Institute of Geography and Statistics (IBGE) estimated that, on July 1, 2014, the older population (over 60 years old) corresponded to about 10% of the adult population (over 18 years old), with older women representing 53.5% of the total older population [9].

### Patients/sample

Individuals with compromises that hindered communication or the understanding of the questions were excluded from the research population. Cluster sampling plans were selected in two stages, Census Enumeration Area (CEA) and household. The selection of the CEAs was made with a probability that was proportional to their number and private households in the 2010 Demographic Census (CD2010) of the IBGE. Households were selected by systematic sampling with random starts and distinct intervals. All older people present in the selected households were interviewed.

### Sampling

The EDOC-I included 1016 older people interviewed. However, 50 subjects of the effective sample did not have HGS measurement, resulting in a sub-sample that had its sample weights corrected and recalibrated to produce estimates for 23,416 older people. Further details of the sampling plan of the EDOC-I, calculation, and calibration of the weights of the sample and subsamples are found in Amaral et al. [9].

### Measurements

Household interviews were conducted with the study participants who answered a structured questionnaire covering socioeconomic, demographic, lifestyle, and health aspects. The trained interviewers evaluated anthropometric measurements and clinical conditions.

#### Dependent variable

Handgrip Strength (HGS) was measured, in kilograms (kg), using a SAEHAN SH5001® brand hydraulic hand dynamometer with a resolution of 2 kg, following procedures adopted by the American Society of Hand Therapists previously showed [10]. Measurements were obtained in standardized conditions, with the participants in the seated position, elbow at 90°, and the handle adjusted to the second position. After receiving explanation of the procedures and after familiarizing then with the instrument, they applied the maximum grip strength for 3 to 5 s. The procedure was performed three times with each hand alternately, with an interval of one minute between each measurement. The maximum HGS was identified considering the highest HGS value among six measures, regardless of the individual hand dominance.

#### Covariates – sociodemographic

Physical activity was analyzed in three aspects: physical activity in displacement - considering active the individual who moved to school or to work walking or using a bicycle whose time to go and return was more than 10 min; occupational physical activity - considering active the subject who reported carrying weights or walking intensely in their jobs or who did the house cleaning alone or, in case of receiving help, being the ones responsible for the most massive part of the cleaning; and physical activity in leisure - considering active those who reported practicing exercises or sports in the last three months with a minimum duration of 150 min per week in the case of moderate activities, or 75 min a week in vigorous activities.

Smoking was initially defined into three categories, including current smokers (those who smoke every day), former smokers (those who used to smoke every day), and non-smokers. Since it was not identified any significant difference between the two first groups, they were aggregated and compared to non-smokers.

Only current alcohol use was considered, although data were also collected on abusive use (binge drinking) in the last 30 days. Due to the low frequency of abusive use reported, the analysis was based on user vs. non-user.

#### Covariates – clinical factors

The investigation of functional independence was based on Katz’s Modified Activities of Daily Living (ADL) scale and the Instrumental Activities of Daily Living (IADL) scale, as described previously [9]. Older people scoring less than five points in the ADL scale was considered as dependent in this analysis, as well as those who needed help for at least one of the activities addressed in the IADL scale.

The Geriatric Depression Scale (GDS-15) was used to assess the presence of depression, considering scores above five points as suggestive of depression [11].

In the definition of morbidities, musculoskeletal complaints were considered based on the self-report of the presence of “much” or “very much” pain in the joints or limbs, in the back, neck or shoulders, or based on reports of diagnoses of arthritis, arthrosis, tendonitis, and repetitive strain injury or osteoporosis. Cardiovascular events were defined by the occurrence of cerebrovascular accident, infarction or angina, heart failure, and arrhythmias or atrial fibrillation. Insomnia (trouble sleeping) was also identified by the self-report of its occurrence (possible answers: “yes” or “already had” or “no”).

Other morbidities were defined based on laboratory test results of blood samples: anemia (hemoglobin ≤13 mg/dL in males, or ≤ 12 mg/dL in females) [12]; diabetes (glycemia ≥126 mg/dL) [13]; hypercholesterolemia (total cholesterol ≥190 mg/dL); altered HDL-cholesterol (< 40 mg/dL in males, or < 50 mg/dL in females), and hypertriglyceridemia (triglycerides ≥150 mg/dL) [14]. The use of medication to control serum levels was also considered.

Dyslipidemia was defined according to the lipid fraction that was altered, triglycerides ≥150 mg/dL, LDL-cholesterol ≥160 mg/dL, HDL-cholesterol < 40 mg/dL in males, or < 50 mg/dL, in females [14].

For the definition of the metabolic syndrome, a combination of the presence of at least three components of glycemia ≥110 mg/dL, systolic blood pressure ≥ 130 mmHg and/or diastolic ≥85 mmHg, triglycerides ≥150 mg/dL, HDL-cholesterol < 40 mg/dL in males, or < 50 mg/dL in females, and abdominal circumference > 102 cm in males, and > 88 cm, in females [15].

Hypertension was identified based on the mean of the second and third blood pressure measurements (systolic blood pressure ≥ 140 mmHg and/or diastolic ≥90 mmHg) or based on the use of hypotensive medication [16].

The waist circumference, waist-to-hip ratio (WHR) and body mass index (BMI) were obtained as the mean of two repeated measures. In the case of waist circumference, measures > 102 cm in men and > 88 cm in women were considered very high. WHR, in turn, was considered high when values were ≥ 1.0 in men and ≥ 0.85 in women [17]. BMI was obtained as the ratio between weight (kg) and the height squared (m^2^). Specific cutoff points for BMI to older people were: BMI < 22 for low weight; BMI between 22 and 27 for eutrophic weight; and BMI > 27 for overweight [18].

### Statistical analysis

Descriptive statistics were obtained focusing on measures of central tendency (mean and median) and 1st quintile of the Maximum HGS stratified by sex and age group (60–69, 70–79, and 80 or over). According to Fried [19], the 20th percentile of the Maximum HGS by sex and age group was adopted for the definition of low HGS and normal HGS, from which a description of the population was made through sociodemographic variables, life habits, and health conditions focusing on measures of absolute (observed n and expanded N for the population) and relative frequency, estimating the differences in the proportions among subjects classified with low HGS by the Pearson Chi-square test.

Thus, the Odds Ratio (logistic regression) models were used to estimate the associations between low HGS and health variables. Bivariate and multivariate analyses by sex estimated the magnitudes of association. The multivariate models, adjusted for covariables (sociodemographic, life habits, and health conditions) that were associated with the outcome of *p* ≤ 0.20 in the bivariate analysis, were defined using the Enter method, with their respective confidence intervals at 95% (95% CI) by the Wald statistic. The models that best fit the data were determined using the Hosmer-Lemeshow test, the Akaike criterion, and the percent concordant. The significance level of 5% was adopted. Besides, in all the analyses the effect of the complex sample design and the weights of the observations were taken into account using the proc. survey routines of the statistical package SAS® version 9.3.

## Results

The mean handgrip strength in older people was 27.2 kg, and the mean difference between one age group and another, for example, 60–69 to 70–79 and 70–79 to ≥80, was − 4.1 kg, being these differences greater among men. The 20th percentile of HGS per age group in males ranged from 28.8 kg in the sexagenarians to 20.8 kg in the octogenarians. Among women, the 20th percentile of HGS ranged from 18.7 kg among 60–69 years old to 12 kg among the oldest women, with a stronger difference between the ages of 70–79 and 80 or more (Table 1).

**Table 1:** Mean, median and 20th percentile of the maximum HGS among older people

Variables	Overall			Men			Women		
	Mean	Median	P20	Mean	Median	P20	Mean	Median	P20
**Total**	**27.2**	**25.0**	**18.9**	**33.6**	**33.1**	**25.0**	**21.6**	**20.6**	**16.8**
Age (age group)									
60–69 years old	29.4	27.4	20.0	36.4	36.6	28.8	23.3	21.9	18.7
70–79 years old	25.5	23.9	18.0	31.5	30.6	24.7	20.4	19.6	16.1
≥ 80 years old	21.3	20.3	15.2	25.9	24.6	20.8	17.4	16.7	12.0

In general, the prevalence of low HGS was 16.7% in men and 17.8% in women. At the intersection of the low HGS with sociodemographic and life habits variables, it was found the association with occupational physical activity in both men and women. There were also borderline associations of the level of HGS with physical activity in leisure among women and smoking among men (Table 2).

**Table 2:** Prevalence of Low HGS by sex, according to sociodemographic characteristics and life habits among older people

Variables	Overall				Men				Women			
	Low HGS (≤P20)				Low HGS (≤P20)				Low HGS (≤P20)			
	n	N	%	***χ***^**2**^	n	N	%	***χ***^**2**^	n	N	%	***χ***^**2**^
**Total**	**169**	**4054**	**17.3**	**–**	**66**	**1822**	**16.7**	**–**	**103**	**2232**	**17.8**	**–**
Age (age group)				0.592				0.751				0.517
60–69 years old	78	2228	16.6		31	1061	16.9		47	1166	16.4	
70–79 years old	58	1168	17,5		21	468	15.2		37	700	19.4	
≥ 80 years old	33	658	19,7		14	292	19.0		19	365	20.4	
Referred skin color				0.705				0.509				0.810
White	43	1023	18.3		18	493	19.7		25	530	17.1	
Non-white	126	3031	17.0		48	1329	15.8		78	1702	18.0	
Marital status ^a^				0.122				0.087				0.587
With partner	53	1323	14.7		28	736	13.7		25	587	16.2	
Without partner	115	2712	19.0		38	1086	20.0		77	1626	18.4	
Education				0.045				0.155				0.137
Illiterate	71	1678	20.7		27	733	19.5		44	944	21.8	
Elementary School	80	1955	17.3		34	960	17.7		46	995	16.9	
High School	18	421	11.0		5	129	8.0		13	293	13.2	
Physical activity in leisure ^a^				0.007				0.100				0.056
Active	6	155	6.9		3	88	7.6		3	67	6.0	
Sedentary	161	3857	18.6		61	1693	17.7		100	2164	19.4	
Occupational Physical Activity or in Displacement				0.007				0.052				0.014
Active	57	1415	13.3		19	531	12.2		38	884	14.1	
Sedentary	112	2638	20.6		47	1291	19.7		65	1347	21.6	
Screen time per day ^a^				0.984				0.974				0.962
Up to 3 h	119	2927	17.1		47	1334	16.3		72	1592	17.8	
More than 3 h	46	1034	17.1		16	413	16.4		30	620	17.7	
Smoking				0,073				0.065				0.417
Non-smoker	20	500	11.7		9	258	9.9		11	242	14.4	
Smoker, ex-smoker	149	3553	18.6		57	1564	18.9		92	1989	18.4	
Alcohol consumption ^a^				0,193				0.234				0.690
No	152	3580	17.8		55	1497	11.0		97	2083	14.7	
Yes	9	255	11.8		6	182	17.4		3	73	18.0	

^a^*missing*; *n* = number of observations in the sample; *N* = population inference based on the sample weights; % = estimated prevalence based on weighted frequencies; χ ^2^ = *p*-value of the Pearson Chi-square test

Regarding the analysis of associations between HGS (low vs. normal) and health/clinic conditions (Table 3), associations with the variables BMI, self-assessment of health in relation to people of the same age, IADL, and anemia were statistically significant in both sexes. Specifically, among women, it was observed that those who had insomnia had a significantly higher proportion of low HGS (23.0%) than those without insomnia (14.4%). Only, among men, the prevalence of low HGS was significantly higher in those who assessed their current health worse than it was 12 months before the interview, those with dependence in ADL, and those with diabetes. Additionally, borderline associations (0.05 < *p* < 0.10) of HGS were identified with self-assessment of health and hypertriglyceridemia, among men, and with metabolic syndrome, among women.

**Table 3:** Prevalence of Low HGS by sex, according to health conditions among older people

Variables	Overall				Men				Women			
	Low HGS (≤P20)				Low HGS (≤P20)				Low HGS (≤P20)			
	n	N	%	***χ***^**2**^	n	N	%	***χ***^**2**^	n	N	%	***χ***^**2**^
Waist circumference ^a^				0.652				0.249				0.956
Normal	106	2627	17.5		57	1561	17.6		49	1066	17.2	
Altered	60	1362	16.4		9	261	13.2		51	1101	17.5	
Waist-to-hip ratio ^a^				0.200				0.629				0.187
Normal	65	1647	15.4		39	1082	15.9		26	565	14.6	
Altered	100	2319	18.5		26	717	18.0		74	1602	18.8	
BMI*				< 0.001				0.035				< 0.001
Low weight	41	926	31.8		16	389	30.9		25	537	32.4	
Eutrophic	56	1400	15.8		26	771	15.9		30	629	15.8	
Overweight	67	1599	14.2		23	625	13.5		44	974	14.6	
Self-assessment of health				0.080				0.077				0.540
Very Good /Good /Regular	135	3231	16.4		54	1483	15.4		81	1749	17.3	
Bad /Very bad	34	822	22.3		12	339	26.6		22	483	20.0	
Assessment of current health in relation to that of the previous 12 months ^a^				0.011				0.006				0.398
Better /Equal	101	2391	15.1		38	1040	13.3		63	1352	16.8	
Worse	68	1662	22.2		28	782	26.0		40	880	19.7	
Assessment of health in comparison with people of the same age ^a^				< 0.001				0.005				0.020
Better /Equal	119	2814	15.2		46	1227	14.1		73	1587	16.2	
Worse	44	1090	27.5		17	507	28.4		27	583	26.8	
Dependence in ADL ^a^				0.035				< 0.001				0.830
No	139	3338	16.3		49	1363	14.5		90	1975	17.9	
Yes	29	692	24.5		17	459	32.4		12	233	16.6	
Dependence on IADL ^a^				< 0.001				< 0.001				0.032
No	59	1492	12.3		24	693	11.3		35	799	13.3	
Yes	109	2537	22.8		42	1129	24.0		67	1408	21.9	
Depression in the GDS*				0.094				0.246				0.269
No	104	2506	15.9		44	1213	15.4		60	1293	20.5	
Yes	64	1514	20.3		21	576	20.1		43	938	16.4	
Insomnia ^a^				0.024				0.347				0.015
No	97	2310	15.0		45	1225	15.5		52	1085	14.4	
Yes	72	1744	21.9		21	597	19.9		51	1147	23.0	
Musculoskeletal complaints				0.733				0.701				0.335
No	70	1722	16.8		41	1099	17.4		29	623	15.7	
Yes	99	2332	17.7		25	723	15.8		74	1609	18.8	
Cardiovascular event				0.203				0.509				0.171
No	125	2992	16.5		50	1368	16.0		75	1625	16.9	
Yes	44	1061	20.2		16	454	19.2		28	607	21.1	
Anemia ^a^				< 0.001				< 0.001				0.022
No	107	2573	13.8		35	1010	11.9		72	1563	15.4	
Yes	56	1355	30.3		30	791	35.1		26	564	25.3	
Diabetes ^a^								0.046				0.191
No	138	3277	17.0		51	1393	15.3		87	1884	18.4	
Yes	28	712	18.1		14	407	24.0		14	305	13.6	
Hypertension ^a^				0.190				0.195				0.733
No	48	1165	20.0		22	585	21.1		26	580	18.9	
Yes	120	2867	16.5		43	1216	15.3		77	1651	17.6	
Hypercholesterolemia ^a^				0.261				0.875				0.107
No	86	2056	18.3		39	1039	16.8		47	1017	20.1	
Yes	78	1891	15.7		26	762	16.3		52	1129	15.3	
Altered HDL cholesterol ^a^				0.090				0.404				0.176
No	105	2579	15.7		45	1269	15.7		60	1311	15.8	
Yes	58	1349	19.8		20	532	19.6		38	817	19.9	
Hypertriglyceridemia ^a^				0.033				0.054				0.287
No	103	2480	19.5		47	1256	19.9		56	1224	19.1	
Yes	62	1491	14.1		18	544	12.0		44	946	15.6	
Dyslipidemia ^a^				0.280				0.265				0.563
No	38	951	20.4		22	595	20.7		16	356	19.9	
Yes	127	3020	16.2		43	1206	15.1		84	1814	17.0	
Metabolic syndrome ^a^				0.080				0.386				0.173
No	91	2213	18.9		35	989	18.5		56	1224	19.2	
Yes	71	1690	14.8		29	790	14.8		42	900	14.9	

^a^missing; *n* = number of observations in the sample; *N* = population inference based on weights and sampling design; % = estimated prevalence based on weighted frequencies; χ^2^ = p-value of the Pearson chi-square test

Table 4 presents the results of the non-adjusted and adjusted logistic regression analyses, identifying the crude and independent effects of the factors associated with the occurrence of low HGS among older men and women. The odds of low HGS were significantly and consistently higher among older people with low weight (men: aOR = 2.80; 95%CI: 1.19, 6.61; women: aOR = 2.61; 95%CI: 1.46, 4.66) and with anemia (men: aOR = 4.15; 95%CI: 2.09, 8.21; women: aOR = 1.80; 95%CI: 1.06,3.06).

**Table 4:** Logistic regression of the low HGS (≤ P20) with independent variables, by sex, among older people

Variables	Men		Women	
	OR (CI95%) Crude	OR (CI95%) Adjusted	OR (CI95%) Crude	OR (CI95%) Adjusted
Marital status (With vs. Without partner)	1.57 (0.91, 2.70)	2.44 (1.32, 4.51)	–	–
Smoking (Smoker /Ex-smoker vs. Non-smoker)	2.11 (0.89, 4.97)	3.25 (1.25, 8.44)	–	–
Assessment of current health in relation to that of the previous 12 months (Worse vs. Better /Equal)	2.28 (1.23, 4.21)	2.21 (1.14, 4.30)	–	–
ADL (Dependent vs. Independent)	2.83 (1.63, 4.93)	2.92 (1.35, 6.30)	–	–
BMI				
Low weight	2.38 (1.07, 5.27)	2.80 (1.19, 6.61)	2.56 (1.49, 4.33)	2.61 (1.46, 4.66)
Eutrophic	1	1	1	1
Overweight	0.83 (0.44, 1.57)	1.09 (0.50, 2.38)	0.91 (0.91, 1.32)	0.86 (0.58, 1.26)
Anemia (Yes vs. No)	4.01 (2.14, 7.51)	4.15 (2.09, 8.21)	1.86 (1.08, 3.22)	1.80 (1.06, 3.06)
Diabetes (Yes vs. No)	1.75 (0.98, 3.12)	1.95 (1.00, 3.81)	0.70 (0.40, 1.23)	0.53 (0.28, 1.01)
Insomnia (Yes vs. No)	–	–	1.77 (1.09, 2.89)	1.83 (1.10, 3.03)
Waist-to-hip ratio (Altered vs. Normal)	–	–	1.35 (0.85, 2.14)	1.79 (1.02, 3.12)
Physical activity in displacement /occupational physical activity (Yes vs. No)	–	–	1.67 (1.09, 2.56)	1.75 (1.08, 2.84)
*p*-value (Wald)		< 0.001		< 0.001
% Concordance		76.0		67.4

% Concordance is a fitness quality criterion of the logistic regression model

The presence of diabetes was found to be significantly associated with low HGS as a risk factor among men (aOR = 1.95; 95%CI: 1.00, 3.81), having a partner (aOR = 2.44; 95%CI: 1.32, 4.51); being a smoker or ex-smoker (aOR = 3.25; 95%CI: 1.25, 8.44); current self-assessment of health as worse than that of the previous 12 months (aOR = 2.21; 95%CI: 1.14, 4.30); and presenting dependence in activities of daily living (aOR = 2.92; CI95%: 1.35, 6.30) were associated with low HGS in men. Among women, there were higher odds of occurrence of low HGS associated with altered WHR (aOR = 1.79; 95%CI: 1.02, 3.12), insomnia (aOR = 1.83; 95%CI: 1.10, 3.03) and physical activity in displacement/occupational physical activity (aOR = 1.75; 95%CI: 1.08, 2.84).

## Discussion

This work confirms the significant decrease of HGS in older men and women, generally indicating a steeper decline in men [4, 20], as well as an intensification of this decline with age in older women. It points out the consistency, in both sexes, of the association of low HGS with low weight and anemia. It also shows the extent to which sociodemographic and behavioral factors, as well as health conditions, are differently associated with low HGS among older men and women.

The option to define the low strength by the lower quintile of the HGS distribution by sex and also by age group (60, 70 and 80 years old or older) was justified by the identification, both among men and women, of a strong correlation of HGS with age [10], which allowed some neutralization of the effect of this variable in the analyses. Differently from the option adopted here, the use of the 20th percentile of HGS adjusted by sex and BMI has been recommended to define low HGS, especially in studies about the frailty phenotype [19]. However, if the 20th percentile of HGS of the entire older population had been adopted, disregarding age ranges, more than 50% of men and women over 80 would be classified as having low HGS, and only a small number of youngest-old would be identified as ‘weak’, which would probably lead to important changes in the results presented here.

BMI is used as a criterion for the definition of nutritional status, and the associations between low weight and low HGS shown here agree with well-established evidence [21]. Hand dynamometry has been recognized as a useful marker of functionality as well as an objective marker of malnutrition [22, 23].

Studies with inpatients revealed that HGS is a reliable measure for the prediction of malnutrition, resulting in more extended hospitalization, clinical complications, and death [23]. Functional losses related to malnutrition can be recovered after protein uptake, and hand dynamometry captures these changes with improved strength levels more rapidly than the BMI [24], which is an alternative for the BMI between the criteria for assessing nutritional status in geriatric patients [25].

Anemia has a multifactorial etiology and, in the older people, contributes to morbidities, physical performance reduction, and the increase in the number of falls, frailty, dementia, hospitalization, and mortality [26]. Identifying the association of HGS with anemia, consistently among men and women, in the general population, ratifies previous findings where reduced hemoglobin levels were linked to low HGS and other criteria of frailty and physical disabilities in the elderly community [27].

This study also confirms that the effect of anemia on the occurrence of low HGS is independent of other sociodemographic, anthropometric, and clinical factors [28]. Although a possible link between reduced HGS and the multiple etiological factors of anemia beyond the scope of the present study is recognized, a plausible explanation for the findings is that the reduction of hemoglobin levels decreases the oxygen consumption capacity of the muscles, leading to tissue hypoxia that promotes the decline of physiological reserve [29].

The relationship between low HGS and diabetes had already been reported in a previous study conducted in Rio Branco among adults aged 18–96, where men with weak HGS presented odds of occurrence of self-reported disease four times higher than those with normal HGS [7]. In this sense, this research contributes to confirming this relationship, considering diabetes diagnosed in the laboratory.

The literature predominantly establishes positive association between the level of HGS and the prevalence of diabetes among men and women [30, 31]. Regarding the association of HGS with the incidence of diabetes, prospective studies are contradictory [32, 33]. The association of HGS with diabetes may be justified by its intimate connection with muscle mass, which plays an important role in the use of blood glucose, also due to its size and responsiveness to insulin [34].

Other explanatory factors of the variation in the occurrence of low HGS had significant effects only in one sex or the other.

Among men, having a partner resulted in a risk factor for low HGS. Although the mechanisms of these associations are unclear, the marital situation is likely a proxy of something that is closely related to HGS, which may reflect accommodation greater preference for household habits, sedentarism, among others. However, in the same sense, it was reported that being married was associated with lower HGS [4] among men aged 72, although a protective effect of marriage on strength has been found among young and middle-aged adults [4, 35]. Studies are needed to explore better the relationships between HGS and marital status in different age groups, since this relationship is little-known.

The smoking history was another factor that was independently associated with the occurrence of low HGS only among men. Smoking had a high prevalence in the study population, especially among women, and the finding that this habit among them did not related to the low HGS is remarkable. Therefore, this is worthy of the exploration in future studies. The relationships of smoking with detrimental health effects are already widely recognized, and it has already been identified in previous studies that smoking men have reduced HGS compared to non-smoking peers [36]. As a possible explanation for the mechanisms underlying muscle reduction among smokers, a review has gathered evidence that the constituents of circulating cigarette smoke seem to play an important role in this process, since they induce loss of muscle mass, reduce oxygen supply, and impair mitochondrial function [37].

The decline in physiological reserve caused by aging leads to a loss of functional independence - a central aspect of the health of older people. The literature has broadly established a relationship between low HGS and dependence in ADL among men and women [38–40] but has been less consistent in establishing the relationship with IADL [39, 40]. In any case, the measurement of HGS as a useful tool in the identification of people at risk of future functional decline has been sustained [41]. The findings of this study only confirm, among men, the association between low HGS and dependence on ADL, regardless of other factors.

Furthermore, the assessment of the current health as worse than 12 previous months ago was relevant to the explanation of the variation in the occurrence of low HGS among men. Although self-assessment of health is an indicator of objective physical and mental health conditions, its use in HGS studies is unknown. However, parallel relations support the consideration of the variable and greater exploitation of its effects in different contexts [42]. It is worth mentioning that this study explored self-assessment with three distinct but correlated indicators, as well as the finding that self-assessment of health is a construct that differs between the sexes, with unequal health profiles of men and women being influenced by their perceptions [20].

Among women, in addition to reduced BMI, the presence of anemia and absence of diabetes were independently associated with increased chances of low HGS, altered WHR score, insomnia, and inadequate physical activity in displacement and/or occupational activity.

WHR is an indirect measure of central adiposity, and the accumulation of visceral fat is responsible for the concentration of inflammatory mediators that can result in sarcopenia and frailty, which may explain the association of altered WHR and reduced HGS among women [43].

Although insomnia was considered based on self-report, its association with low HGS finds resonance in other studies [44, 45], although it has presented a relation only among women. A recent study with middle-aged and older people demonstrated a quadratic relationship in which both reduced hours of sleep in both sexes and excessive sleep in women were associated with a steeper decline in HGS over four years of follow-up. One possible explanation on the relationship between sleep and HGS is in the circadian clock, where sleep hours and sleep quality act in musculoskeletal physiology, regulating and being influenced by sleep, by genetic mechanisms and inflammatory processes, which are also associated with loss of strength [45].

Among the limitations of the present research, it must be emphasized a possible attenuation of the associations due to the survival effect. Also, the punctual diagnosis of the diseases, both those defined by physical examinations, such as hypertension, and those resulting from clinical laboratory tests from blood dosage that could falsify results, was somehow mitigated by broad guidance on the protocols to carry out the examinations and evaluations. On the other hand, as a strong point of this study, it should be noted that its results are inferential to the older population of the capital of the state of Acre and that laboratory and clinical measures were used to define the diseases, which allowed to inform people who were unaware of the presence of certain diseases, either by limitation or lack of access to health services or even by a lack of awareness of the need for such care.

## Conclusion

Factors associated with low HGS are not equal between the sexes. Among older men, low HGS was associated with low weight (BMI), anemia, diabetes, having a partner, having smoking history, negative self-assessment of current health compared to the previous 12 months, and dependence in ADL. Among older women, low HGS was associated with low weight (BMI), anemia, diabetes (surprisingly, a protective effect was observed here), altered waist-to-hip ratio, insomnia, and insufficient physical activity in displacement or occupational tasks.

The findings reinforce HGS as a health biomarker in older people of both sexes and support its use as a viable strategy which can easily be incorporated into both rehabilitation and primary health care, not only for the screening of the low strength as an indicator of health problems in older people but as a measure, along with other criteria, for monitoring health throughout life, thus allowing promising early intervention actions in disease prevention and health promotion.

## Data Availability

The datasets used for this study are available from the corresponding author upon reasonable request.

## Abbreviations

IADL: Instrumental Activities of Daily Living
ADL: Activities of Daily Living
DC: Demographic Census
GDS: Geriatric Depression Scale
EDOC-I: Study of Chronic Diseases - Older People
HGS: Handgrip Strength
HDL-cholesterol: High Density Lipoproteins-Cholesterol
IBGE: Brazilian Institute of Geography and Statistics
CI: Confidence Interval
BMI: Body Mass Index
OR: Odds Ratio
aOR: Adjusted Odds Ratio
WHR: Waist-to-Hip Ratio
χ2: Pearson’s Chi-square test
